# A review of the United Arab Emirates healthcare systems on medical tourism and accreditation

**DOI:** 10.3389/frhs.2024.1329252

**Published:** 2024-02-21

**Authors:** Amna I. Alshamsi

**Affiliations:** Occupational Health-Psychology and Management, Quality Department, Emirates Health Services, Dubai, United Arab Emirates

**Keywords:** hospital accreditation, medical tourism, United Arab Emirates, healthcare sector, quality and safety

## Abstract

This paper aims to review the healthcare system in the United Arab Emirates (UAE) and the utilisation of international accreditation to improve the quality of healthcare services and to grow its medical tourism industry. Medical tourism has contributed to the UAE's economy. Hence, the country mandated international accreditation in public and private facilities to attract patients and boost medical tourism. Accreditation is recognised worldwide as one of the main drivers for implementing quality and patient safety standards, which has sparked considerable interest in studying the effects and outcomes of such assessments. Therefore, the second aim of this paper is to review the UAE's strategic goals to improve the quality of healthcare services using international accreditation. Although striving to achieve global accreditation attracts tourists, it is essential to understand the needs and outcomes of such assessments. This review showed how the UAE healthcare sector has evolved to improve the quality of its healthcare services through accreditation. While enhancing the quality of such services and increasing the medical tourism industry provided many opportunities for expatriates to move to the UAE, the country should aim to strengthen its medical services by expanding to other Middle Eastern countries. This paper could influence policymakers implementing international accreditation in the UAE and the Middle Eastern region.

## Introduction

Quality and patients' safety are key elements in any healthcare systems. Significant strides forward have been observed in reforming healthcare facilities to improve and sustain quality of care through accreditation assessments. Such improvement was accelerated by the call from the Institute of Medicine's report, “*To Err is Human”*, which highlighted the errors healthcare professionals (HCPs) often made at work (1). Accreditation is recognised worldwide as one of the main derivers for implementing quality and patients' safety standards. Therefore, many countries, including the United Arab of Emirate (UAE), mandated accreditation to ensure quality and safety of health services. Accreditation has sparked a considerable interest in studying the effect and impact of these assessment. The findings of previous literature however have lacked consistency and robust evidence to support the accreditation of healthcare organisations. Therefore, the current paper aims to review the UAE's strategic goals to improve the quality of healthcare services and grow its medical tourism industry.

### The healthcare system in the UAE

The UAE has a unique demographic structure, as most of its population is made of expatriates who have moved to the country to take up work in the service sector. The growth of the service sector has increased the number of people moving to the UAE. For instance, Melodena (2) showed that 82% of Dubai's population are expatriates. In 2016, the total estimated population of UAE was 9.3 million, which has increased to 10.1 million in January 2022 (3). The rapid growth of the UAE's population and its economic condition has positively influenced the healthcare system. Furthermore, the number of healthcare facilities and healthcare workers has increased to meet the growing demand of UAE's population as a result (4). In 2014, a set of strategic goals were launched by the UAE government to achieve a global leading country focussing on services as a key contributor to the growth of the country, and improving the healthcare system is one of these objectives.

The UAE has a relatively robust public healthcare system, which include the ministry of health and prevention (MOHAP) and a developing private sector. MOHAP is a centralised management organisation that oversees healthcare facilities nationwide; however, each emirate regulates its local facilities to manage and allocate resources. Other public healthcare systems in the UAE include the Health Authority of Abu Dhabi (HAAD), Dubai Health Authority (DHA), and the Emirates Health Services (EHS). While Abu Dhabi and Dubai have their own authorities, EHS operates as a regulating and licensing authority for the remaining five emirates. Despite the advancement of the UAE's health system, there is a growing need for healthcare professionals (HCPs) to ensure adequate staffing and overcome the constant demand, which arises with the provision of high-quality healthcare services. Since 2009, the number of hospitals has increased by 25% with a total of 36 government and 79 private hospitals (4). Government facilities provide free care for UAE nationals and a low-cost option for non-nationals. Given the high cost of private facilities, new amendments were made to include health insurance allowing residents to access healthcare facilities at a low cost and minimise the pressure on public hospitals (5). As a result, such amendments have attracted tourists and medical staff to specialised hospitals in the country.

While the UAE has managed to develop a robust healthcare system, the country faces some inherent disadvantages. For instance, most healthcare workers are expatriates, risking the availability of HCPs in the future. It is claimed that there are 181 doctors per 100,000 residents (5). Therefore, attracting internationally qualified practitioners and increasing the number of HCPs (i.e., doctors and nurses) is a top priority is a top priority for the UAE. Due to its dependence on expatriate HCPs, retaining qualified workers is a key challenge for the country. Based on the WHO's World Statistics (6), the UAE was reported to have a shortage in nurses and midwives. For instance, an estimate of only 31 nurses and midwives were assigned for every 100,000 residents in the UAE compared to the UK with an estimate of 88 nurses and midwives were assigned for every 100,000 residents.

The possible shortage in HCPs could lead to undesirable consequences in sustaining the UAE's vision in improving the quality of healthcare services. Further, the psychological health and well-being of HCPs could possibly be affected due to the increase in work demands. Similar challenges were found in other countries; for example, the continuous growth of population and chronic diseases had led to an insufficient number of HCPs, which was proved to be a major concern in the United States health system (6). Furthermore, the international migration of healthcare workers from low-income to high-income nations has been observed (7, 8). As noted by Thompson and Walton-Roberts (7), the immigration of trained nurses influences the quality of services, suggesting that countries should aim to provide access to quality training to retain healthcare workers. To overcome the possible shortage of HCP, the government of UAE developed a new policy to provide a 10-year visa to HCP, which was perceived positively (8).

On the other hand, healthcare has a significant impact on boosting the growth of the economy in the UAE. The national vision embraced the expansion of the medical tourism sector and attracting healthcare professionals as well as patients. To attract patients and boost medical tourism, UAE's health sector requires improving the quality of healthcare services and promoting patient safety. Hence, the country has witnessed a substantial growth in the number of the accredited facilities. The following section discusses aspects of medical tourism and its impact on the UAE healthcare inspections and accreditation.

#### Medical tourism in the UAE

“Medical Tourism” refers to the movement of patients who travel for medical services to gain a rapid access to healthcare services with reduced costs or a higher quality of care (9). Medical tourism has long been a factor in the growth of the economies of many developed countries. The relationship between travelling and health is not new as people used to travel for wellness spas and resorts. Indeed, patients are motivated to travel to different countries to seek treatment because of the availability of a desired care, the cost of such care, and/or the expertise of medical staff providing the care.

Furthermore, profits related to medical tourism are rapidly increasing, with an estimated annual revenue of $50–$65 billion, and this is expected to continue to grow by 15%–20% annually (10). Undoubtedly, this growth can be explained by the additional services patients receive besides their medical treatment, including accommodation, transportation, and post-treatment vacations (11). As a result, developing countries, such as the UAE, aspiring to become regional centres for health tourism (12), have been competing intensely with each other to attract patients and secure their place in the industry. Despite the possible risks of malpractice and life-threatening complications, governments and policy makers continue to invite patients for state-of-the-art health services including one-day surgeries.

Asian countries, such as Thailand, have attracted patients from the US for low-cost healthcare. Middle Eastern patients, nevertheless, have travelled for services that are not available in their countries, for instance advanced or complicated treatments, such as cancer treatment (11). The concept of medical tourism is not new, however; it has been redefined by globalisation. From the UAE, patients traditionally travelled to India, Thailand, or Singapore for medical care. Woodman (13), published a guide for patients (Patients Beyond Borders) on how to choose appropriate countries for care. The author reassured patients that they would be pleased by the quality of care provided in these countries because they offer “affordable, high-quality and American-accredited medical options” (13). Medical tourism is therefore a major contributing factor in the growth of international accreditation programmes to assess the quality of healthcare services in the UAE.

Seeking treatment in an accredited facility has become a criterion for patients to travel abroad. Accordingly, countries such as Turkey, Saudi Arabia, and the UAE have raced to accredit their facilities with international accrediting bodies, to maintain their place in medical tourism (14, 15). One of the largest accrediting organisations is the Joint Commission (JC), which extended its services to survey primarily hospitals and primary care centres in the US (16), with an estimate of 1,000 healthcare organisations being accredited worldwide (17). The JC extended its services since 2000 to accredit international healthcare organisation using the Joint Commission International (JCI) standards.

In 2014, Khan and Alam (15) published a paper showing which countries had healthcare facilities accredited by the JCI and comparing the totals. The UAE had 39 facilities accredited by the JCI. Since then, a sharp increase in the number of accredited facilities has been observed in the UAE. Data was taken from the JCI official website (www.jointcommissioninternational.org), which showed a total of 214 facilities accredited by the JCI in 2023. Besides, there is a substantial increase in the number of facilities accredited by the JCI since 2013, with the UAE taking the lead.

The increase in accredited facilities in the UAE supports the visons of developing countries aspiring to boost their medical tourism (14, 18). It is worth mentioning that, in the UAE, accreditation is mandatory for healthcare facilities including hospitals, primary healthcare centres, laboratories, and dental centres. Hence, the UAE has the highest number of accredited facilities.

### Healthcare accreditation

Accreditation is recognised worldwide as one of the main derivers for implementing quality and patients' safety standards. It is a voluntary external process that evaluates healthcare organisations based on a set of standards carried out by healthcare professionals (19). External surveys and accreditation in healthcare facilities were developed to ensure objectivity and scrutiny of assessments (19). Generally, the process of inspection or assessment by an international organisation is referred to as the survey and inspectors are called surveyors (20). The process of accreditation resembles a peer review assessment given that surveyors are carefully selected to match the clinical quality of the surveyed facility. Standards, however, are developed to include various services provided in healthcare organisations through a consensus process (21).

The Institute of Medicine's report, “*To Err is Human”* highlighted the errors healthcare professionals (HCPs) often made at work (1). The report, urged healthcare organisations to create a safe environment for patients through regulatory mechanisms that include accreditation, licencing, and certification. The World Health Organization has also supported the establishment of national programmes that encourage the continuity of healthcare improvement and evaluate healthcare organisations' performance (21, 22). As a result, many countries developed strategies to utilise accreditation to ensure best practices are being fulfilled in health organisations which have limited resources, including quality and patients' safety.

Since the 1990s, a rapid growth in evaluating healthcare systems has been observed, which was triggered by the need to know how to enhance the delivery of services with the least possible resources (23). Data published by the Office for National Statistics (24) showed that the UK, in 2017, spent around £3,000 per individual on healthcare. Further, the percentage of growth domestic product (GDP) spent on healthcare had fallen from 9.8% in 2013 to 9.6% in 2017. This compares with 8.6% of budget allocated to the healthcare in the UAE in 2017, which had fallen to 6.9% in 2020 (25). However, the Organisation for Economic Co-operation and Development reported an increase in healthcare spending worldwide on average of 2.0% (26).

Therefore, countries endorsed strategies to reduce spending on healthcare systems through implementing concepts of quality and standardisation (i.e., Accreditation). It is not clear whether accreditation is designed to help reduce spending on healthcare. However, looking at figures from the UAE's budget allocated to healthcare and the number of accredited facilities (i.e., 214), one might consider a correlation between accreditation and the percentage of spending on healthcare.

#### Accreditation in the UAE

As mentioned earlier, the UAE has a comprehensive public healthcare system. Improving the quality of healthcare has always been a priority among UAE's health authorities (25). According to the World Health Statistics series (27), the UAE showed promising trends in promoting the health of residents by eradicating infectious diseases, such as malaria and measles. Further, in 2017, the maternal mortality ratio (MMR) was reported to be 6 per 100,000 live births, compared to 14 in the US, and 9 in the UK (27).

In 2009, MOHAP announced that healthcare organisations, public or private, should aim to implement systems to meet international standards in healthcare quality and patients' safety. Accreditation was voluntary at that time. In 2014, a set of strategic goals were launched by the UAE government to achieve a world class healthcare system by 2021. These strategies were part of the UAE's national agenda, which was launched by the president of the UAE and supported by his vice president (28). Since these declarations, healthcare organisations, public and private, started to implement international standards, mainly the JCI standards, for accreditation. Such declaration has led the UAE to have the highest number of JCI-accredited organisations, which was recognised by the President and Chief Executive officer of the Joint Commission for its achievement (29).

Indeed, implementing international standards across the UAE helps to improve the quality of care and patient safety; however, earlier studies showed that the process also can lead to increased stress for HCPs. Yet, few empirical research has been conducted to examine the effect of JCI accreditation in UAE's healthcare facilities. Devkaran and O'Farrell published several studies on the impact of accreditation in a private hospital in Abu Dhabi (i.e., the capital of the UAE) (12, 30, 31). In their first study, the authors recorded a series of 23 quality measures monthly for 4 years. The measures were related to the JCI standards, which included but were not limited to the assessment of patients. Findings showed that, although measures of performance had fallen immediately after the accreditation inspection, these measures thereafter remained steady and sustained improvement until the end of accreditation cycle, i.e., 3 years.

Devkaran and Farrell (12) hypothesised a four-phase cycle model predicting HCPs' compliance with accreditation standards. The first phase is the initiation phase consisting of a slow improvement and gradual compliance with the standards. The second phase starts three to six months before inspectors visit and a sharp improvement occurs in facilities. The authors suggest that in this phase employees reach the peak of compliance, and everyone works hard to achieve the accreditation. The third phase, which appears immediately after the assessment is finished, shows a drop in the level of compliance. The fourth phase, which is the inactivity phase, is where compliance to standards is flattened. In a follow up study, Devkaran, O'Farrell (31) evaluated the hospital performance after three cycles of accreditation using 27 quality measures. Their results suggested that the level of compliance among HCPs was maintained. They also explained how achieving the first cycle of accreditation with a high level of quality was important to maintain this level of quality.

Further, Koornneef, Robben and Blair (4) reviewed peer-reviewed publication on the progress of the healthcare system in the UAE and the challenges that healthcare organisations face. Interestingly, and although the UAE has the highest number of JCI-accredited organisations, several studies reported on the quality and outcome of accreditation and concluded that accreditation did not contribute to the overall improvement. Therefore, there is an ongoing need to investigate the impact and outcomes of hospital accreditation on both HCPs and the working environment in healthcare settings.

Other authors studied the impact of accreditation on the stress level of HCPs in the UAE (32–35). For example, Alshamsi, Thomson and Santos (32) interviewed HCPs to understand their perceptions with the accreditation process. Their finding showed that HCPs perceived the process of accreditation to increase their stress level, on one hand. On the other hand, support from colleagues and leaders were perceived to increase HCPs work engagement. The authors extended their research to test the effect of work-related factors [i.e., Job Demands-Resources (36)] before accreditation on HCPs burnout and work engagement level after accreditation (35). Their results showed that demands before accreditation predicted burnout level among HCPs after accreditation.

## Discussion

Although the impact and outcome of accreditation in healthcare organisations remain debatable, the UAE mandates such assessments to regulate the quality of healthcare services. The international accreditation of public and private facilities attracted patients to the UAE and boosted medical tourism. Significant strides have been observed in reforming healthcare facilities to improve and sustain the quality of care through accreditation assessments. Accreditation has sparked considerable interest in studying the effect and impact of this assessment. The findings of previous literature, however, need more consistency and robust evidence to support the accreditation of healthcare organisations. To sustain quality of healthcare services, it was suggested to introduce long-term training programs that correlate medical tourism with accreditation (37).

Furthermore, the UAE has been recognised as a safe and respectful country. Therefore, the country needs to advertise more about medical tourism internationally and locally. The government should initiate and sustain campaigns for the safety and quality of healthcare services. The government can also propose medical tourism products that classify healthcare services based on patient reviews concerning quality and safety. Finally, the UAE must be aware of medical tourism's emphasis on tourism's politics, culture, and social identities and its impact on the country and the region.

In conclusion, this review discusses impact of accreditation in the United Arab of Emirates (UAE) context. The review showed how the UAE healthcare sector has evolved to improve the quality of healthcare services through accreditation. While the development of health medical industry in the UAE provided many opportunities of expatriates to move to the UAE, the country should aim to strengthen its medical services by expanding to other Middle Eastern countries. Furthermore, the UAE should assess the impact of accreditation on the HCPs as previous studies demonstrated how accreditation demands influenced the mental health of HCPs (33, 35). Given that burnout among HCPs can reduce the quality of care, it is essential for the UAE government to acknowledge such an impact by developing robust regulations and guidelines to promote a healthy working environment for HCPs during accreditation. Furthermore, this review raises intriguing questions regarding the role of accreditation in the UAE in promoting the tourism industry.
